# Modulation of transcription factor dynamics allows versatile information transmission

**DOI:** 10.1038/s41598-023-29539-3

**Published:** 2023-02-14

**Authors:** Alan Givré, Alejandro Colman-Lerner, Silvina Ponce Dawson

**Affiliations:** 1 Departamento de Física, Facultad de Ciencias Exactas y Naturales, Universidad de Buenos Aires, Buenos Aires, C1428EGA Argentina; 2 Instituto de Física de Buenos Aires (IFIBA), CONICET-UBA, Buenos Aires, C1428EGA Argentina; 3 Departamento de Fisiología, Biología Molecular y Celular, Facultad de Ciencias Exactas y Naturales, Universidad de Buenos Aires, Buenos Aires, C1428EGA Argentina; 4 Instituto de Fisiología, Biología Molecular y Neurociencias (IFIBYNE), CONICET-Universidad de Buenos Aires, Facultad de Ciencias Exactas y Naturales, Buenos Aires, C1428EGA Argentina

**Keywords:** Cell signalling, Transcription, Computer modelling, Information theory

## Abstract

Cells detect changes in their environment and generate responses, often involving changes in gene expression. In this paper we use information theory and a simple transcription model to analyze whether the resulting gene expression serves to identify extracellular stimuli and assess their intensity when they are encoded in the amplitude, duration or frequency of pulses of a transcription factor’s nuclear concentration (or activation state). We find, for all cases, that about three ranges of input strengths can be distinguished and that maximum information transmission occurs for fast and high activation threshold promoters. The three input modulation modes differ in the sensitivity to changes in the promoters parameters. Frequency modulation is the most sensitive and duration modulation, the least. This is key for signal identification: there are promoter parameters that yield a relatively high information transmission for duration or amplitude modulation and a much smaller value for frequency modulation. The reverse situation cannot be found with a single promoter transcription model. Thus, pulses of transcription factors can selectively activate the “frequency-tuned” promoter while prolonged nuclear accumulation would activate promoters of all three modes simultaneously. Frequency modulation is therefore highly selective and better suited than the other encoding modes for signal identification without requiring other mediators of the transduction process.

## Introduction

Living organisms react to changes in their environment. The signaling systems that are used to “interpret” these changes and generate end responses are ubiquitous: they operate in both bacteria and eukaryotes and in processes as diverse as bacterial chemotaxis or the maturation of the immunse system, among many others. The malfunction of the information transmission systems involved in these processes is the cause of various pathologies. For this reason, understanding the way that living systems process and transmit information is fundamental from both a basic and applied point of view. The generation of responses to external stimuli usually involves changes in the intracellular concentration of some intermediaries which often induce changes in the nuclear concentration of transcription factors (TF) and, consequently, in gene expression. Commonly, the intensity of the stimulus is encoded in the *amplitude* of this concentration, but in others in the time it remains at a high level (*duration*) and recently it became apparent that in other cases the stimulus causes pulses of nuclear accumulation, the *frequency* of which correlates with stimulus intensity. Examples of these different strategies are abundant in many cell types^1^. In yeast 10 TFs^2^ show pulsatile nuclear localization. For example, the TF Crz1 responds to a rise in extracellular calcium with stereotyped pulses of nuclear accumulation, the frequency of which positively correlates with the extracellular calcium concentration^3^. The case of Msn2 is perhaps more surprising, since its behavior depends on the stimulus: it responds to glucose limitation with pulses of nuclear accumulation of increasing frequency the lower the glucose concentration, to osmotic stress with an initial burst of nuclear localization the duration of which depends on the strength of the osmotic shock, and to oxidative stress with a prolonged nuclear localization of amplitude (the fraction of nuclear Msn2) proportional to the magnitude of the stress^4–6^. In mammalian cells, the transcription factor NF-$\kappa$B responds to a variety of stimuli with pulses of nuclear accumulation. In this case, the strength of the stimulus does not affect the frequency but the amplitude of the nuclear pulses^7^. Finally, the mammalian tumor suppressor p53 is another TF that responds differently depending on the stimulus. While UV exposure elicits a single pulse of increasing amplitude and duration with increasing UV dose, pulse sequences are elicited upon double-strand DNA breaks caused by $\gamma$-radiation with the number of pulses increasing with the level of damage^8^. Are there any advantages associated to a particular type of codification? When is one of these modes better suited than the other? These are two motivating questions of the present work.

The examples just described show that different external stimuli can be encoded, transmitted and decoded by common signaling components^9–11^ and that different end responses can be elicited depending on the dynamics of TFs nuclear concentrations. For example, p53 pulses induce the expression of DNA repair genes while a single sustained p53 pulse leads to the expression of senescence genes^12^. Something similar happens with the TFs, NF-kB^13–15^. The TF, Msn2, which participates in the regulation of the multi-stress response in yeast^16,17^, is a good motivating example for the studies of the present paper given the different dynamics it displays depending on the stimulus^4^ which suggest that Msn2-regulated promoters of genes induced by glucose deprivation (like DCS2^18^ and HXK1^19^) are activated by *frequency modulation*, that promoters of genes induced during osmotic stress (e.g., SIP18^20,21^ and ALD3^22,23^) are activated by *duration modulation*^24^ and that those of genes induced by oxidative stress are activated by *amplitude modulation*. Following the works of O’Shea’s group^4,24,25^ we may say that the type of codification identifies the type of stimulus. The question then arises as to how the promoters involved in these responses, which are regulated by the same TF, differ from one another so as to be induced by one or another type of modulation. This is another motivating question of the present study which, as the previous ones, can be addressed within the framework of information theory^26,27^. This approach has been used to analyze cell signaling and infer properties of the underlying network of interactions, both from a theoretical point of view^28,29^ or using experimental data^25,30,31^. The information-based description involves defining what constitutes the input, the output and the channel connecting them. Using information theory it is possible to quantify the extent to which the output “distinguishes” the values that the input can take on. Given the distribution of input values, this is quantified by the *mutual information* between input and output, which is usually measured in bits, with *n* bits corresponding to distinguishing $2^n$ (sets of) input values. In the case of the cell, the ability to distinguish different inputs is key to generate adequate responses to each situation.

In this paper we analyze the mutual information between stimulus and response when the channel involves encoding the stimulus strength in either the amplitude, the frequency or the duration of a TF’s nuclear fraction. To this end we use the simple model introduced by Hansen and O’Shea to describe the activity of Msn2, defining as the input the amplitude, duration or frequency of the TF in the nucleus and, as output, the accumulated amount of mRNA produced, which correlates well with protein expression^24^. To account for stochastic fluctuations, which are not negligible in biological processes and can limit the information capacity of cell signaling pathways^30,32,33^, we not only model the transcription step stochastically using a Markov process but also include noise in the amplitude of the TF concentration and in its interpulse frequency. Hansen and O’Shea^30^ applied information theory to quantify the gene expression information transduced by Msn2 in yeast using experimental data obtained with high-throughput microfluidics. Instead, our approach is theoretical and seeks to determine the largest mutual information that can be achieved depending on the stimulus strength encoding. In addition, we ask whether there are disjoint regions of optimal parameter values for each encoding type that could allow the use of a single TF to elicit different end responses depending on the encoding. We found that, in most cases, the maximum possible mutual information is $\sim$ 1.5 to 1.8 bits, which is slightly larger than the values estimated from experiments in wild type yeast cells but similar to those obtained in mutants^25^. As discussed later, this value can be improved depending on the timing of the end response generation. We also found that the information transmitted, irrespective of the mode of encoding, is overall higher as the threshold of TF concentration needed for gene expression is higher or when promoter activation occurs on a faster timescale. Of the three modes, we found that frequency encoding is the most sensitive to changes in the kinetic parameters, while duration enconding is the least sensitive. This explains the experimental results of Hansen and O’Shea^25^ and suggests that the cell can realize dynamic multiplexing by using a gene that transmits a large amount of information for the least sensitive modulation (duration) and a much smaller amount through the highly-sensitive channel (frequency).

## Methods

### Model, inputs and outputs

**Figure 1:**
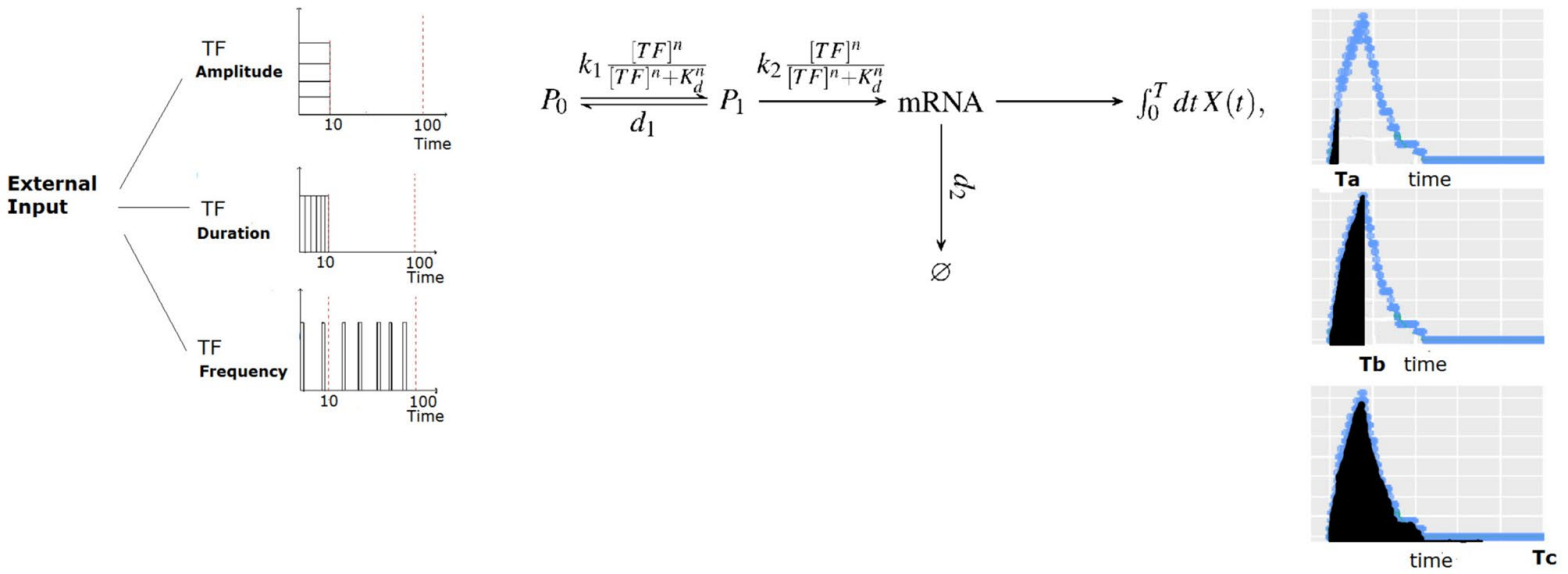
The model. An external stimulus is encoded in the (pulse) duration, (pulse) amplitude or (inter-pulse) frequency of the nuclear *TF* concentration, [*TF*]. The model does not describe this first step of the codification process, but starts directly with the nuclear *TF* time course. Mutual information, *MI*, between input (pulse duration, pulse amplitude or mean inter-pulse frequency, depending on the type of stimulus codification or “*TF* modulation type”; 3 subfigures on the left) and output (the time integral of the number of mRNA molecules produced, *X*) is computed choosing three possible time cuts for its calculation (3 subfigures on the right which correspond to an example with a single TF pulse of amplitude, 100, and 10 min duration). In between, converting input into output, is the transcription model^24^ described in more detail in the main body of the paper.

We consider the simplified transcription model^24^ depicted in Fig. 1. In this scheme, *TF* is the (nuclear) transcription factor which is assumed to undergo a known dynamics (piecewise constant in time). As explained later in more detail, the arbitrary concentration units that are used throughout the paper are such that 100 corresponds to the maximum possible value of nuclear [*TF*]. $P_0(t)$ and $P_1(t)$ represent the promoter in its inactive or active state, respectively ($P_0+P_1=1$). The promoter is activated by *TF* in a cooperative fashion, as reflected in the term, ${[TF]^n}/({[TF]^n+K_d^n})$, where *n* indicates the cooperativity and $K_d$ represents an effective dissociation constant, measured in the same arbitrary units as [*TF*], of the binding/unbinding reaction which is assumed to occur on a faster timescale than the rest of the processes and, therefore, be in equilibrium. The *TF*-bound promoter then becomes active with rate, $k_1$. The arrow from $P_1$ to mRNA represents transcription which occurs at rate, $k_2{[TF]^n}/({[TF]^n+K_d^n})$, meaning that *TF* needs to be bound for transcription to take place. More details on the model can be found in the Supplementary Note. Given that $0\le [TF]^n/({[TF]^n+K_d^n})<1$, $k_1$ and $k_2$ are, respectively, the maximum rates at which promoter activation and transcription occur. The model includes mRNA degradation at rate, $d_2$, but not the translation into the protein which is assumed to decay slowly enough so that the accumulated amount of mRNA produced can be used as the output of the process.

In the simulations, transcription is modeled stochastically with a master equation, while the rest of the steps are modeled deterministically, using Euler’s method to solve the ODEs. The *TF*’s nuclear concentration, [*TF*](*t*), is modeled by a single or a sequence of square pulses. The maximum *TF* amplitude considered is 100 (in dimensionless units) and the maximum pulse duration is 10 min. The total time of the simulations is 100 min. The value, $P_1(t)$, derived from the numerical integration of the first two steps and [*TF*](*t*) are fed into the transcription Markov Process where the core of the randomness occurs. Finally, the mRNA is integrated in time to obtain the output. Given that $P_o+P_1=1$, the simulations imply solving the following equations:

1 $$\begin{aligned} {\dot{P}}_1=\frac{k_1 [TF(t)]^n}{K_d^n+[TF(t)]^n}-\left( \frac{k_1 [TF(t)]^n}{K_d^n+[TF(t)]^n}+d_1\right) P_1,\quad X=N\xrightarrow []{\frac{{k_2} [TF(t)]^n}{K_d^n+ [TF(t)]^n}P_1(t)}{X=N+1},\quad {X=N}\xrightarrow []{d_2 X}X=N-1 , \end{aligned}$$

where *X*(*t*) is the (stochastic) number of mRNA molecules at time, *t* and $N=\{0,1,2\ldots \}$.

Three types of *TF* modulations are studied: duration (identified with the label “0”), amplitude (identified with the label “1”) and frequency (identified with the label “2”). In the cases of modulation by duration or amplitude, *TF*(*t*) is given by a single pulse of amplitude, 100, and variable duration or of variable amplitude and duration, 10 min, respectively, that starts at $t=0$. Neglecting the noise that is added to the concentration amplitude, $[TF]=100$ is the maximum value that this concentration can take on for all cases probed. In the case of frequency modulation, *TF* pulses of 1 min duration and amplitude, 100, that occur all throughout the simulation are considered with a Poisson process to determine their timing. In all cases, *TF*(*t*) is the norm of the sum of the deterministic pulse amplitude at time *t* and a normally distributed random variable of standard deviation, 10. The output is:

2 $$\begin{aligned} Out({T}) = \int _0^T dt \, X(t), \end{aligned}$$

with, in most cases, one of three finite values, $T=T_{a}$, $T_{b}$ and $T_{c}$. These values are $T_{a}=10$ min, $T_b=50$ min and $T_c=100$ min and $T_a=1$ min, $T_b=10$ min and $T_c=100$ min for frequency and amplitude modulation, respectively. For the latter, $T_b$ corresponds to the time at which the *TF* pulse ends for all amplitudes. For duration modulation, we use $T_a=t_{end}$, $T_b=10$ min and $T_c=100$ min, with $t_{end}$ the duration of the input *TF* pulse. Notice that the $T_a$ time cut of this case is qualitatively different from the other 8 cuts, since it does not correspond to a fixed time, but instead it is defined by an event. This makes the promoters selected to be sensitive to that particular setting different from the others. For illustrative purposes we also compute, *Out*(*T*), and the corresponding mutual information, as continuous functions of time, *T*.

### Computation of mutual information

Discretizing the set of values that the input, *I*, and the output, *O*, can take on ($\{I_i\}_{i=1}^{N_I}$ and $\{O_i\}_{i=1}^{N_O}$, respectively), their mutual information can be written as:

3 $$\begin{aligned} MI(I,O)&=-\sum _{i=1}^{N_I} p_I(I_i) \log _2\left( p_I(I_i)\right) -\sum _{i=1}^{N_O} p_O(O_i) \log _2\left( p_O(O_i)\right) +\sum _{i=1}^{N_I}\sum _{j=1}^{N_O} p_{IO}(I_i,O_j)\log _2\left( p_{IO}(I_i,O_j)\right) , \end{aligned}$$

where $p_{I,O}$ is the joint probability distribution of *I* and *O* and $p_I$ and $p_O$ are the corresponding marginal distributions. All the simulations are done assuming a uniform distribution of the input values: over [1, 10] min for duration modulation; over [0, 100] for amplitude modulation; over (0, 0.1]/min for frequency modulation. The highest frequency considered gives, on average, a time integral of [*TF*](*t*), over the simulation time interval (100 min), that is equal to the equivalent integrals for the cases of amplitude or duration modulation ($100\times 10$ min). The uniform distribution implies that $p_I(I_i)=1/N_I$ $\forall i$, so that the first term in the r.h.s. of Eq. (3) is equal to $\log _2(N_I)$. All the simulations are done discretizing the corresponding input in $N_I=200$ values. Given the input parameters that are kept fixed depending on the modulation type (pulse amplitude and/or duration) and a set of model parameters ($k_1$, $k_2$, *n*, $K_d$, $d_1$ and $d_2$), 15,000 simulations are run for each of the 200 input values that correspond to the modulation type probed (amplitude, duration or mean inter-pulse frequency). The results of these 3$\, 10^{6}$ simulations are then used to compute the 3 types of output cuts described before (which only differ in the time interval over which the number of mRNA molecules is integrated, see Eq. (2)). The mutual information between each of the three input types and each of the three output cuts (9 combinations) is computed, for each set of kinetic parameter values, using the Jackknife method, which corrects for undersampling^34,35^. For some studies we also compute *MI* as a function of time, in which case we call it *MI*(time). We compute *MI*(time) for each input type integrating *X* in Eq. (2) between 0 and time.

### Sampling of parameter space, optimization and comparisons.

One of the aims of the present study is to determine the parameters of the model that maximize *MI* for each input modulation and output time cut. To guarantee a homogeneous sampling of the parameter space, the Latin Hypersquare Sampling (LHS) method was used^36^, dividing the logarithmic range of values of each parameter ($k_1,k_2,K_d,n,d_1$) into equiprobable, non-overlapping intervals^37^. The intervals sampled for each parameter are shown in Table 1. In total, 17,500 parameter sets were probed.

**Table 1:** Sampled intervals for each of the parameters of the problem.

Parameter	Range
$k_1$ (1/min)	(0.01, 1)
$k_2$ (1/min)	(1, 100)
*n*	(1,10)
$K_d$ (a.u.)	(10, 100)
$d_1$ (1/min)	(0.01, 1)
$d_2$ (1/min)	0.12

To obtain the results of the subsection “One transcription factor, two genes” we look for the set of parameters that gives the smallest value, $MI_m$, for one type of modulation and output time cut, *i* ($i=0,1,2$ as described before) restricting the search over the sets of parameters that give values, *MI*, that are within 90% of the maximum, $MI_M$, for another type of modulation ($j\ne i$) and the same time cut. We then define the *Minmax* region of parameters for the corresponding pair of input types, *ij*, as the sets that give values of *MI* that differ by less than 10% from $MI_M$ and $MI_m$ for the *i* and *j* input modulation types, respectively. We also say that the sets of parameters in the *Minmax* fulfill the *Minmax* condition.

## Results

To analyze the mutual information (*MI*) between a stimulus and its induced transcriptional response, and how *MI* depends on whether stimulus strength is encoded in amplitude, frequency or duration of a transcription factor activation state, we performed simulations of a simple transcriptional model first presented by Hansen et al. to describe gene induction by the TF Msn2 of the budding yeast *S. cerevisiae*^24^. As illustrated in Fig. 1, we assumed that increasing stimulus strengths could modulate nuclear TF (*TF*, the input of our model) in three possible ways: larger *TF* concentrations (amplitude modulation), longer periods of maximal *TF* concentration (duration), and higher frequency of *TF* pulses (frequency). The output (transcription) was obtained integrating the number of mRNAs produced over three different time intervals. TFs perform two tasks: binding to DNA and when bound, regulating transcription itself. This is captured in the model by two consecutive steps. In the first step of the model, binding of the TF to the promoter facilitates its transition from inactive ($P_0$) to active ($P_1$). That is, the TF makes the promoter permissible for transcription. This reaction, modeled as a cooperative Hill function, aggregates in one step various processes besides binding, such as modifications in the positioning of the nucleosomes and recruitment of chromatin modifying enzymes. In the second step of the model, the bound TF induces transcription, capturing in it several other molecular steps by which the TF brings to the proximity of the promoter the complexes that actually recruit and activate the RNA polymerase II, depending on the promoter, either the Mediator or SAGA complexes^38^. TF recruitment of this machinery is likely to be cooperative as well, and thus it is modeled by a second Hill function (see the Supplementary Note for more details). In the model, each set of parameter values of the transcription model corresponds to a different promoter. In this section, we show the results obtained when *MI* was computed for each combination of input modulation and output time cut. In some instances, we also show the results of computing *MI* as a continuous function of the output time cut. To interpret the results, we analyze the time course of some key variables of the model. We study as well how *MI* varies with the parameters of the model and whether there are sets for which *MI* is large for an input modulation type and much smaller for another.

### Maximum mutual information and model parameters

Here we show the results of the optimization run for each combination of input modulation type and output time cut when the parameters, $d_1$, $k_1$, $k_2$, $K_d$ and *n*, are varied and $d_2=0.12 /$min (see “Methods”). Table 2 displays the parameter sets that maximize *MI* in each case and the range of values over which the optimization was performed. We observe that $MI_M$ is 1-2 bits for all combinations (with a value $\sim \log _2(3)$, i.e., three distinguishable input values), with the exception of the $T_a$ output time cut and duration modulated inputs that gives 2.6. It must be pointed out that in this case the output is integrated only while *TF*(*t*) is different from 0 (while the pulse is on), which is qualitatively different from the rest. For the other cases, the system can behave slightly better than a binary (noisy) switch (1 bit). The parameters that maximize *MI* have various features in common for almost all combinations of input modulation and output time cut. If we set aside the output cut, $T_a$, for duration modulation, we observe that $k_2\gg k_1, d_1, d_2$, implying that the timescale of transcription must be as fast as possible to guarantee a good information transmission. Second, $n\ge 3$ in most cases and the dissociation constant of the *TF* binding/unbinding reaction is $K_d\gtrsim 50$, of the same order of magnitude but smaller than the maximum *TF* concentration (100). The lower bound, 50, is also 5 times larger than the noise amplitude that is considered in the model (10). *MI* attains its maximum for $k_1\gg d_1$, a condition that guarantees that, at steady state, $P_1\approx {[TF]^n}/({[TF]^n+K_d^n})$. Thus, provided that $k_1\gg d_1$, the ability to distinguish different amplitudes that range between 0 and 100 will depend on how the function ${[TF]^n}/({[TF]^n+K_d^n})$ maps the [0, 100] interval.

**Table 2:** Sets of parameters that maximize mutual information, *MI*, for each combination of input modulation and output integration time and the corresponding value, $MI_M$, obtained in each case.

Input	Output	${k_1}$ [1/min]	${k_2 }$[1/min]	*n* [a.u.]	${K_d}$ [a.u.]	${d_1}$ [1/min]	${ MI_M}$ [bits]
Modulation	Cut	(0.01,1)	(1,100)	(1,10)	(10,100)	(0.01,1)	
Duration	$T_a$	0.32	91	1	10	0.056	2.61
Duration	$T_b$	0.6	84	7.3	79	0.017	1.61
Duration	$T_c$	0.13	63	8.3	94	0.016	1.52
Amplitude	$T_a$	0.62	92	9.5	49	0.024	1.65
Amplitude	$T_b$	0.93	31	3.3	96	0.13	1.91
Amplitude	$T_c$	0.72	45	4.2	81	0.84	1.66
Frequency	$T_a$	0.77	26	9.9	100	0.031	0.82
Frequency	$T_b$	0.08	45	6.5	55	0.012	1.52
Frequency	$T_c$	0.13	35	7.3	54	0.017	1.87

The table shows the range over which each parameter value was varied when looking for this maximum. The parameter, $d_2$, was always fixed at $d_2=0.12/$min. Note that the arbitrary concentration units are such that 100 is the (active) nuclear TF concentration that was used to compute *MI* in the case of duration or frequency modulation and the maximum such concentration in the case of amplitude modulation.

**Figure 2:**
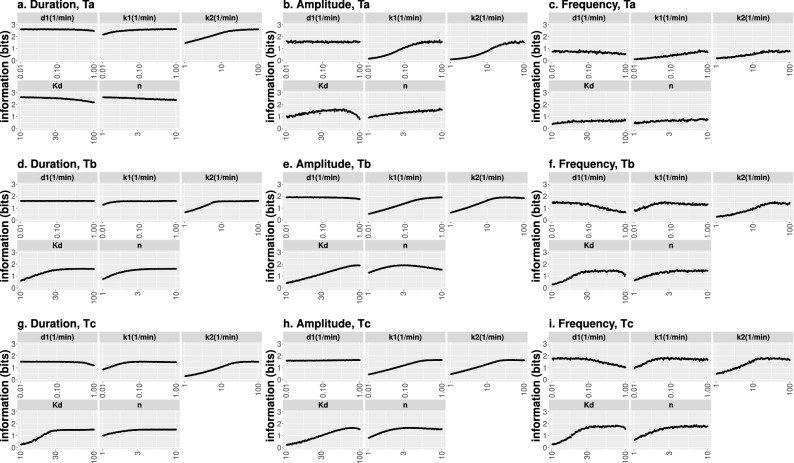
Behavior of mutual information, *MI*, around its maximum value, $MI_M$, when one kinetic parameter is varied. We show *MI* as a function of the corresponding parameter for each type of input modulation and output time cut (**a**–**c**,**d**–**f**,**g**–**i** correspond, respectively, to the $T_a$, $T_b$ and $T_c$ output time cuts; **a**–**d**–**g**,**b**–**e**–**h**,**c**–**f**–**i** correspond, respectively, to duration, amplitude and frequency input modulation type). Except for the modulation by duration and the $T_a$ time cut, the rest of the behaviors are very similar across modulations and time cuts. In particular, the highest information is obtained if $d_1$ is low, $k_1$, $k_2$ and *n* are high and $K_d$ is in a middle-high range, but not too high. For the $T_a$ time cut and duration modulation type $K_d$ and *n* need to be low to maximize *MI*.

To analyze in more detail the effect of each parameter on *MI* we performed a sensitivity analysis varying each parameter while keeping the others fixed at the values that gave $MI_M$. We show the results in Fig. 2. This figure confirms the initial conclusions that we drew from Table 2. Namely, for all but one case (the duration modulation type for the output time cut, $T_a$), $MI_M$ is attained when $d_1$ is low and $k_1$ and $k_2$ are high, compared with the fixed timescale of the model, $d_2=0.12/$min. If we look at the parameters related to the *TF*-promoter relationship, we observe that $K_d$ tends to be medium-high and the Hill Coefficient, *n*, tends to be high for the maximum *MI* to occur. On the other hand, we observe that the range over which the parameters can be varied without changing *MI* much is different ddepending on the parameter and the input modulation. In particular, we observe that amplitude modulation transmission is the most sensitive to separate changes in $K_d$ and *n*, that frequency modulation is most sensitive to changes in $d_1$ while *MI* barely changes upon variations in $d_1$ for duration modulation. In most cases, once the model parameters exceed a threshold, *MI* stays approximately constant and we observe, on average, the most extended plateaus for duration modulation.

### Model dynamics and *MI* maximizing parameters

In order to understand why the maximum information transmission is obtained, in most cases, for parameters that satisfy $k_2\gg k_1, d_1, d_2$; $n\ge 3$ and $K_d\gtrsim 50$, we look at how the time course of some key variables of the model (*TF*, $P_1$, mRNA and *Out*(*time*)) changes depending on whether the parameters are such that they give a relatively large or low value of *MI* for some combination of input type and output time cut.

**Figure 3:**
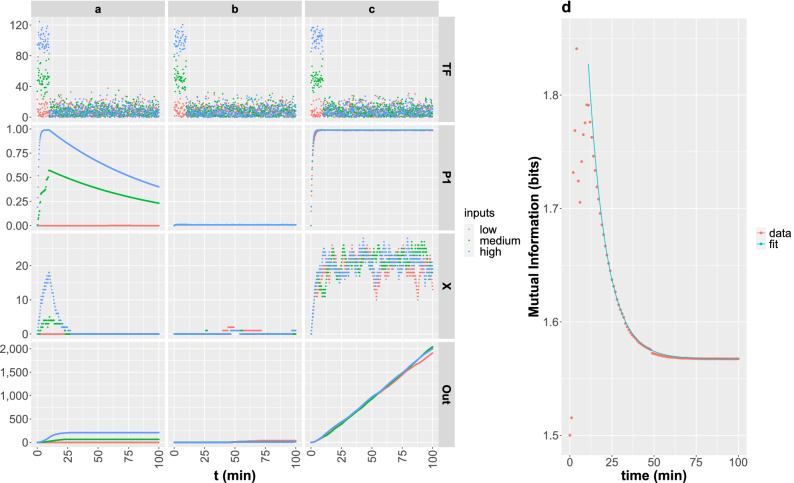
Time course of [*TF*], $P_1$, *X* and *Out*(*time*) for 3 sets of parameter values (**a**–**c**) and 3 different amplitudes of *TF*(*t*) (colors) and the time course of *MI* for amplitude modulation, the $T_c$ time cut and using the kinetic parameters of the example in **a** (**d**). (**a**–**c**) In all the simulations, a single *TF* pulse of 10 min duration was considered. The amplitudes were 0 (red), 10 (green) and 100 (blue) with a superimposed Gaussian distributed noise amplitude of standard deviation, 10. The parameters used in the simulations of the first column were $k1=1/$min, $k2=100/$min, $d1=0.01/$min, $Kd=70$, $n=10$ for which the mutual information, computed for the amplitude modulation input type and the output time cut, $T_c$, is $MI=1.56$ (i.e. $94\%$ of $MI_M$). Those of the second column were $k1=0.01/$min, $k2=1/$min, $d1=1/$min, $Kd=10$, $n=1$ for which $MI=0.03$, and those of the third: $k1=1/$min, $k2=100/$min, $d1=0.01/$min, $Kd=10$, $n=1$ for which $MI=0.02$. (**d**) *MI* as a function of time computed for amplitude modulated inputs and the $T_c$ time cut using the kinetic parameters of the example in a (symbols) and a curve of the form ${\mathscr {A}}\exp (-\lambda (t-10 \,\min))+MI_a$ that fits the eventual decay to its asymptotic value $\sim MI_a=1.57$ bits with $\lambda =0.09/$min (solid line).

We show in Fig. 3a–c, the results obtained with simulations in which a single *TF* pulse of 10 min duration was considered. When the parameters satisfy $k_2\gg k_1, d_1, d_2$, $n\ge 3$ and $K_d\gtrsim 50$ we observe that the three input strengths translate into three distinguishable outputs (Fig. 3a). In the other examples, either promoter activation dynamics is too slow and it never gets activated (Fig. 3b, where $k_1\ll d_1$) or the promoter is activated maximally for all inputs (Fig. 3c, where the threshold determined by $K_d$ and *n* is too low and the timescale is not slow). In the last case, the three input strengths translate into almost indistinguishable outputs. We show in Fig. 3d, with symbols, the time course of *MI* computed for amplitude modulation and the $T_c$ output time cut using the kinetic parameters of the example in a. We observe that *MI* reaches a maximum approximately at $t=10$ min, i.e., when the *TF* pulses end, and then decays towards a slightly lower value at a rate $\lambda \sim 0.09/$min of the order of the mRNA degradation rate, $d_2=0.12/$min, as illustrated by the fitting curve depicted with a solid line in the figure. We obtain a similar non-monotonic temporal behavior of *MI* for duration modulation while for frequency modulation *MI* increases monotonically, i.e. the more time allowed to the system to process the input, the more information can be extracted (data not shown).

### Pairs of parameters and mutual information

**Figure 4:**
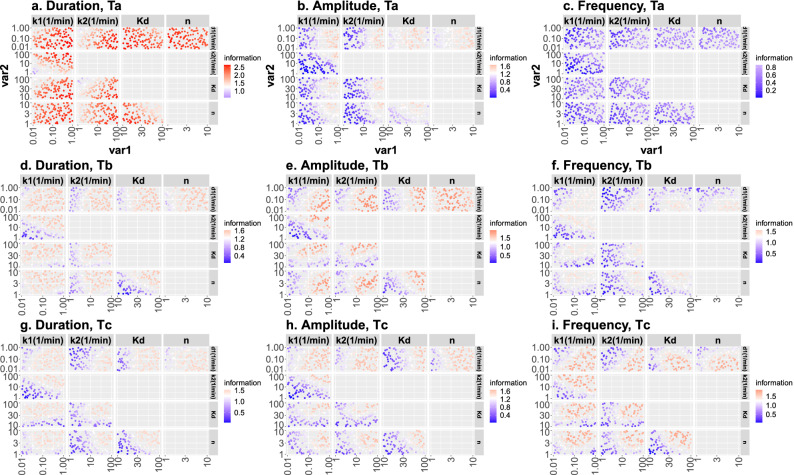
Mutual information, *MI*, as a function of two parameters while the others are left fixed at the values that maximize *MI* for the particular input and output types analyzed in each case (**a**–**c**,**d**–**f**,**g**–**i** correspond, respectively, to the $T_a$, $T_b$ and $T_c$ output time cuts; **a**–**d**–**g**,**b**–**e**–**h**,**c**–**f**–**i** correspond, respectively, to duration, amplitude and frequency input modulation type). Common behaviors are observed throughout most modulations and time cuts.

So far, we have analyzed how *MI* varies when a single parameter is varied. In this section, we analyze if the parameters are interdependent in determining $MI_M$. To this end, we varied the values of all possible pairs of parameters maintaning the others fixed at the values that gave $MI_M$ and calculated *MI* (Fig. 4). From the analysis of the results, we determined how the pairs had to be varied to keep *MI* constant. We found that, for all input and output types, $d_1$ tended to be positively correlated with $k_1$ and $k_2$. That is, changing $d_1$ in a given direction modifies *MI* in a way that is compensated by modifying $k_1$ or $k_2$ in the same direction. We then found that $k_1$ and $k_2$ were negatively correlated with one another, i.e., if $k_1$ is increased, $k_2$ has to be increased to keep *MI* fixed, and viceversa. Similarly, the parameters related to the *TF*-promoter relationship, $K_d$ and *n*, are negatively correlated in most cases, with the exception of the $T_a$ output time cut for duration modulation, for which *n* and $K_d$ need to be low for *MI* to attain its maximum value.

### One transcription factor, two genes

One of the aims of the present work is to determine whether there can be two promoters, modulated by the same *TF*, each one being able to discriminate stimulus strengths encoded in a different property of the nuclear *TF* concentration and not being able to discriminate the strengths encoded in the property at which the other one is good. For example, one promoter that discriminates TF amplitudes but is “blind” to frequency changes while the reverse situation is valid for a second promoter. To explore this possibility, we searched for the sets of kinetic parameters that gave *MI* within 90% of the maximum, $MI_M$, for a certain input modulation type and, at the same time, gave a relatively small value for another input type (and the same output time cut), i.e., the sets that fulfill the *Minmax* condition (see “Methods”).

**Figure 5:**
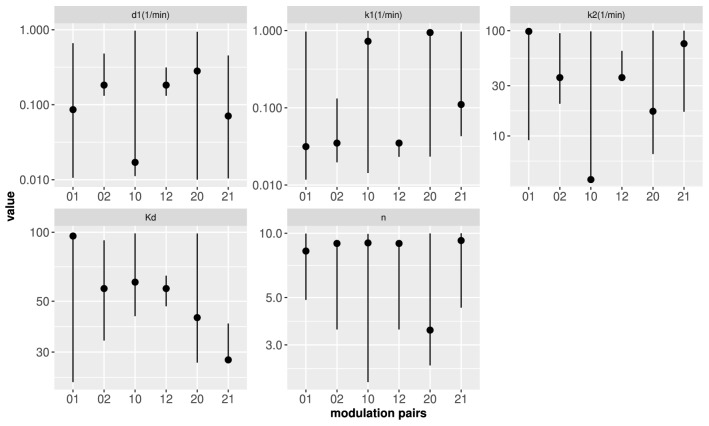
Projection on each parameter axis of the *Minmax* regions obtained for each pair of modulation input types and the $T_c$ output time cut. The pairs of digits in the horizontal axes identify the modes for which the information transmission has been maximized (first digit) and subsequently minimized (second digit) to determine the *Minmax* regions, with 0: duration, 1: amplitude and 2: frequency. The symbols correspond to the values that minimize *MI* for the second digit modulation given that *MI* stays within 90% of $MI_M$ for the first digit modulation.

Figure 5 shows the projection of the six *Minmax* regions, for the $T_c$ time cut, on each of the five parameter space axes of the transcription model. Although such a projection should be analyzed with care, Fig. 5 illustrates how much each parameter can be varied while satisfying the *Minmax* condition. We can observe that the sensitivity of *MI* to parameter variations is different depending on the parameter and the pair of input modulation types. For example, $k_1$ and $d_1$ can be varied by over an order of magnitude and, yet, *MI* differs by less than 10% from $MI_M$ for duration modulation and from the conditional minimum, $MI_m$, for amplitude modulation, and *viceversa*. The parameter, $d_1$, on the other hand, can barely be varied to keep *MI* within 90% of $MI_M$ for amplitude or duration modulation and differ by less than 10% from $MI_m$ for frequency modulation. Overall, we see that keeping *MI* as small as possible for frequency modulation in each *i*2 *Minmax* region requires to fine tune the time-related parameter values ($k_1$, $k_2$ and $d_1$) and, in the case of the 12 region, the dissociation constant, $K_d$, as well. As we show in what follows, this fine tuning allows the finding of promoters (characterized by sets of parameter values) that are “blind” to frequency modulated inputs but are good at transmitting information in other modulation types, while the reverse seems to be impossible.

**Figure 6:**
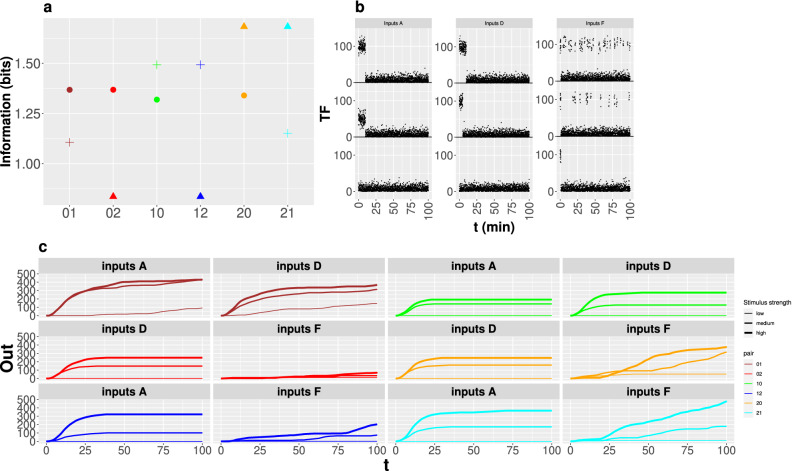
Mutual information and dynamical behaviors obtained using the set in each *Minmax* region that minmizes *MI* for the “second” modulation type. (**a**) *MI* computed using the selected set of parameters in each *Minmax* region for the input modulation modes that are maximized and conditionally minimized to determine the region (circles: duration, crosses: amplitude, triangles: frequency) and the $T_c$ output time cut. The labels on the horizontal axis identify the *Minmax* regions with the digits order as in Fig. 5. Each parameter set is identified by a different color. The parameter values in each set are displayed with symbols in Fig. 5b,c. Accumulated mRNA (*Out*(time)) as a function of time (**c**) obtained from simulations of the model using the *TF* time courses displayed in (**b**) (curves in **c** are plotted with increasing thickness with stimulus strength) and the same parameter sets (identified by the same color) as in (**a**).

We now analyze the difference in information transmission that is achieved when choosing some of the model parameters determined with the *Minmax* conditioning. This is illustrated in Fig. 6a where we show the *MI* values obtained for the *i* and *j* input modes using a parameter set in the *ij* *Minmax* region, for all six *ij* pairs. The parameters are those that minimize *MI* for one input modulation type while still being within $90\%$ of the other’s maximum (plotted with circles in Fig. 5). We observe that *MI*, for duration modulation, has approximately the same value ($\sim$ 1.3 to 1.4) for all combinations, including those for which *MI* by duration is minimized. Given that each set of parameters corresponds to a different promoter, this implies that durations are always equally discriminated regardless of the promoter. In contrast, frequency modulation is the most sensitive mode with *MI* variations of almost one bit depending on the parameters that characterize the promoter ($MI\sim \, 0.8$ for the sets depicted in red or blue and $MI\sim \, 1.7$ for those depicted in orange or cyan). The situation for amplitude modulation is intermediate with variations of $\sim \, 0.5$ bits ($MI\sim \, 1.1$ for the parameters depicted in brown or cyan while $MI\sim \, 1.5$ for those in green or blue). These results indicate that it should be possible to find promoters that, being regulated by one TF, are good at decoding duration or amplitude encoded inputs and, at the same time, be “blind” to frequency modulated ones, but that the separation of behaviors would not be as clear in the opposite situation, especially in the case of the combination frequency-duration. We now analyze how the discriminating ability is reflected in the amount of mRNA that is produced using some examples (Fig. 6).

We show in Fig. 6c, the mRNA that is accumulated as a function of time for various examples that were obtained using the parameter sets of Fig. 6a. We probed the dynamics of the model using these sets of parameters (each one of which can be associated with a different promoter that responds to the same *TF*) and sets of inputs, *TF*(*t*)-D, A, F, characterized, respectively, by decreasing duration, amplitude and frequency of the *TF* pulse or pulses, as shown in Fig. 6b, related to the transmission modes that had been maximized and conditionally minimized to determine the parameter sets.

Consistent with the previous discussion (Fig. 6a), it is the frequency encoding (Inputs F) the one that produces the largest differences depending on the parameter set. Not only the amount of mRNA produced for Inputs F is much larger for the two sets in which transmission by frequency modulation was maximized (20 and 21 *Minmax* regions) than in those in which it was conditionally minimized (02 and 12), but also the three *TF* time courses within Inputs F are more easily distinguishable in the former than in the last two cases. In the case of duration encoding (Inputs D), the amounts of accumulated mRNA are slightly larger for the set that belongs to the 01 *Minmax* region than for the one that belongs to the 10 one and slightly larger for the latter in comparison with the ones in the 02 or the 20 *Minmax* regions. The ability to distinguish the 3 durations does not seem to vary much with the parameter set. In the case of amplitude encoding (Inputs A), the amount of accumulated mRNA is largest for the set which was determined by conditionally minimizing the information transmission for amplitude modulation. For this type of inputs, the failure to transmit enough information for the parameter set in the 01 *Minmax* region compared with the set in the 10 one (see brown *vs* green cross in Fig. 6a) seems to be related to producing similar amounts of mRNA regardless of the *TF* amplitude in the former (see brown *vs* green curves for Inputs A). Something similar occurs when the behavior obtained for Inputs A and the sets in the 12 and the 21 *Minmax* regions are compared (blue and cyan curves of Inputs A, respectively).

## Discussion and conclusions

In this paper we have studied the information transmission capabilities of transcription when extracellular stimuli are encoded in the amplitude, duration or pulse frequency of the TF’s nuclear concentration. We used the simple model of Fig. 1-introduced by Hansen and O’Shea^24^ to describe the activity of Msn2 in yeast- in which each promoter that is regulated by the same TF corresponds to a different set of parameter values of the transcription step. We computed the mutual information (*MI*) between the amplitude, the pulse frequency or the duration of the TF’s nuclear concentration (the input) and the accumulated amount of mRNA produced (the output) to determine both the maximum *MI* for each encoding and the range of parameter values (i.e., the type of promoters) that gives the maximum in each case. Our studies showed that, for any combination of physiologically feasible parameter values and any modulation input type, the maximum *MI* is between one and two bits (Table 2), with the only exception of the $T_a$ output time cut and duration modulation, which is qualitatively different from the rest and for which it could be $\sim \, 2.6$ bits. The maximum values obtained for the other cases were slightly larger than those encountered in experiments on Msn2-regulated gene expression in wild type (WT) yeast cells^25^ which showed that it operated as a noisy switch (transmitting $\sim$ 1.1 to 1.3 bits) for two genes, *HXK1* and of *SIP18*, when they were modulated, respectively, by frequency and amplitude. When the authors mutated the promoters with the aim of improving information transmission, *MI* for amplitude modulation increased to $\sim \, 1.5$ bits^25^, very similar to the maximum values that we found with our parameter exploration. Looking at the time dependence of *MI* for the parameters that gave a relatively good information transmission we observed that, for duration and amplitude modulation, *MI*(*t*) reached a maximum and then decayed to a slightly smaller asymptotic value. For these modulation types, the timing of the maximum and the timescale of the subsequent decay of *MI* are related to the moment at which mRNA production ends and to the mRNA degradation rate, $d_2$, respectively. This is illustrated in Fig. 3d that corresponds to a case in which all pulses end at $t=10$ min and $d_2=0.12/$min, and where it can be observed that the decay to the asymptotic value can be approximated by an expression of the form ${\mathscr {A}}\exp (-\lambda (t-10\,\min ))+1.57$ bits with with $\lambda =0.09/$min. The non-monotonic behavior of *MI* with time is probably the reason that underlies the much larger *MI* value obtained for duration modulation and the $T_a$ output time cut which corresponds to the end of mRNA production in all cases and, thus, does not include the subsequent mRNA degradation steps (included in all other output time cuts). It is also consistent with the observation that *MI* increases steadily with time for frequency modulation, given that, in this case, the TF pulses occurred thoughout the simulation time. Perhaps, cells could take advantage of the non-monotonic time course of *MI* via a pre-equilibrium sensing mechanism^39^, something that we have not explored in the present paper.

The model we have used is characterized by four parameters directly associated to timescales ($k_1$, $k_2$, $d_1$ and $d_2$, which we kept fixed at $d_2=0.12/$min) and by another two ($K_d$ and *n*) related to the *TF*-promoter relationship. We found (Table 2) that those that maximized *MI* for all combinations of input modulations and output time cuts corresponded to a fast transcription timescale ($k_2\gg k_1, d_1, d_2$ with $k_1\gg d_1$ in most cases). This is so because a high transcription rate (at constant mRNA degradation rate, $d_2$) creates ample and input-sensitive mRNA fluctuations (Fig. 3). This result agrees with those of Hansen and O’Shea^24^ in that a fast timescale generates better responses. Regarding the *TF*-promoter relationship, again we found a distinguishing result for the $T_a$ time cut and duration modulation input. In particular, we found that a larger dynamic range (lower $K_d$ and *n*) was favored in this case compared to the others. Restricting the comparison to the *MI* maximizing parameters for the $T_c$ output time cut, we obtained the sharpest *TF*-promoter relationship ($n\sim$ 7 to 8) for duration and frequency modulated inputs and values of $K_d$ that were several times larger than the noise amplitude, 10, and varied between 50% (for frequency modulation) and 94% (for duration modulation) of the maximum *TF* concentration, 100. We suspect these relatively large $K_d$ values (i.e., low binding affinity) are important to prevent the occurrence of noise-driven transcription events (a limit case of which is illustrated in Fig. 3c). Relatively low affinity is thus a condition to guarantee a clear distinction between “ON” and “OFF” states and it is in agreement with the fact that functional low affinity binding sites are common in eukaryotes perhaps to better distinguish between similar TFs^40^. We discuss later why frequency modulation requires a somewhat higher binding affinity than the other two modes. The *n* values that maximize *MI* are also relatively high and might seem unrealistic if only *TF*-DNA binding is considered. In our simplified model, however, both the first and second step synthesize an aggregate of elementary reactions/processes. A series of cooperative reactions, each having a modest $n > 1$ may combine to elicit a sufficiently high combined *n*. There are multiple examples of this sharpening effect in concatenated covalent modification cycles, such as protein kinase cascades^41,42^. As discussed later, the parameter values that yield the absolute maximum, $MI_M$ should be considered with care in that some of them may be varied without producing much change in *MI* (Figs. 2, 4). For example, when *n* is varied between 3 and 10, *MI* varies by less than 10% for all input modulation types and the $T_c$ time cut (Fig. 2g–i). In any case, our studies on the *MI* maximizing parameters should be helpful when designing synthetic promoters. Some of the properties required to maximize *MI* (Table 2) agree with, whereas others seem to differ from, the experimental results of Hansen and O’Shea^24^. Namely, these authors classified Msn2-activated promoters as high (H) or low (L) threshold (requiring high or low Msn2 concentration for induction, respectively, which corresponds to high or low $K_d$) and as fast (F) or slow (S) (induced quickly or requiring a longer time with Msn2 bound to induce transcription, respectively). Their experiments showed that high treshold traits were accompanied by slow timescales (*HS promoters*) and that these genes were those modulated by duration (i.e., osmotic stress). They found, in turn, that low treshold traits were coupled to fast timescales (*LF promoters*) and that these genes were those modulated by frequency (i.e., induced during glucose starvation). While (for the $T_c$ output time cut) we found, in agreement with these results, the maximum *MI* at a larger $K_d$ (i.e. a larger threshold) for the duration modulation than for the frequency one, for the transcription timescale (determined by the ratio, $\frac{k_1 k_2}{k_1+d_1}$) we found, differently from the experimental observations, a value twice as large for duration modulation compared to frequency modulation.

We can explain the differences between the optimal values derived from our study and those calculated with experimental data obtained from WT cells by Hansen and O’Shea^25^ in terms of the different sensitivity that *MI* displays to parameter variations for the various input modulations. In general, once the model parameters surpass a threshold, *MI* stays approximately constant (Fig. 2). On the other hand, in most cases the threshold for one parameter depends on the other parameter values, e.g., the $k_2$ threshold decreases for increasing $k_1$ (Fig. 4). Thus, the “optimal” parameters are not that meaningful per se, in the sense that they could be varied (especially, in pairs) without much change in *MI*. Our study showed that the range over which the parameters could be varied without changing *MI* much was different for the different parameters and input modulation types. For example, for the $T_c$ time cut we obtained that amplitude modulation transmission was most sensitive to changes in $K_d$ and *n*, that *MI* for frequency modulation varied by over half a bit if $d_1$ or $k_1$ did not stay within the same order of magnitude as the values that gave maximum transmission for this mode and that duration modulation was the least sensitive to simultaneous variations in $d_1$ and $k_1$ (Figs. 2, 4). This different sensitivity was clearly reflected in the results of the search of parameters that could give good transmission for one input modulation and a relatively low one for another (Figs. 5, 6a). Namely, we determined that frequency modulation was so sensitive to parameter changes that promoters could be found which yielded a relatively large information transmission for duration or amplitude modulation and a much lower one for frequency modulated inputs: one which gave $MI\sim \, 1.4$ bits for duration modulation and $MI\sim \,0.8$ bits for frequency modulation (Fig. 6a, red symbols) and another which gave $MI\sim \, 1.5$ bits for amplitude modulation and $MI\sim \,0.8$ bits for frequency modulation (Fig. 6a, blue symbols). We observe in Fig. 5 that the parameters that characterize these examples differ from those that yield higher *MI* values for frequency modulation, among other things, in the values of the transition rate from the inactive to the active conformation, $k_1$, and of the dissociation constant, $K_d$: while $K_d\sim \, 60$ and $k_1\sim \, 0.02/$min for the cases that yield $MI\sim \, 0.8$ bits (red and blue triangles in Fig. 6a), it is $k_1>0.1/$min and $K_d\sim$ 30 to 40, in the cases for which $MI\sim \, 1.7$ bits (orange and cyan triangles in Fig. 6a). Higher binding affinities and faster transition rates contribute to transcription initiation during the relatively short pulse duration of frequency modulated inputs. This agrees with the observation that p53 binding affinity to proapoptotic genes is lower than that to proarrest genes^43^ and that p53 pulses lead cells to recover from DNA damage while a sustained p53 elevation frequently lead to senescence^12^. Thus, to some extent we conclude that frequency encoding is selective not only because it requires a relatively fine tuning of the promoter parameters but also because it works well with higher binding affinities.

Having a promoter such that $MI\sim \,1.5$ bits for amplitude modulation and $MI\sim \,0.8$ bits for frequency modulation (blue symbols in Fig. 6a) is similar to the situation encountered by Hansen and O’Shea^25^ using a mutated version of the promoter, SIP18, that is induced upon prolonged nuclear accumulation of Msn2 (see their Fig. 2). Expanding our search beyond the limits of the already defined *Minmax* regions, we found a set of parameters (i.e., a promoter) which gave $MI\sim$ 1.1 bits for duration, $MI\sim$ 1.2 bits for amplitude and $MI\sim$ 0.7 bits for frequency modulation. The last two values are closer to those obtained for SIP18 in WT cells. We think that the information transmission for frequency modulation could have been reduced even further had we used a more sophisticated (and realistic) model than the very simple one of Fig. 1. Hansen and O’Shea^25^ asked why the cell should not “fine-tune the expression level of stress genes to the stress intensity”. Our studies seem to indicate that, at least in the case of SIP18, the parameters might have been tuned to make it as blind as possible for frequency modulation without losing the capability of distinguishing ON from OFF without error in the case of prolonged nuclear accumulation of Msn2. The lower sensitivity to parameter variations of duration and amplitude modulated inputs might have prevented this total blindness to be attained for the HXK1 promoter which is physiologically induced by Msn2 pulses. In any case, according to our studies, even under optimal promoter parameters the information transmission could only be slightly better than 1bit, i.e., each single promoter cannot act as a rheostat. External stimuli encoded in the amplitude or duration of a transcription factor’s nuclear fraction, which can eventually activate more than one promoter (provided that the detection thresholds are surpassed), could increase their information transmission capabilities with a combinatorial strategy. These results are applicable to other signaling pathways. In particular, similar processes might underlie dynamic multiplexing in gene expression within the p53 regulatory pathway in humans^12^, something that could be investigated introducing mutations that could change the activation threshold or characteristic timescales of the promoters involved.

## Supplementary Information

Supplementary Information.

## Data Availability

The codes and the datasets generated and analyzed during the current study are available in the Mendeley Data repository, https://data.mendeley.com/, DOI: 10.17632/8hsddbfgp5.1.
