# Supplementary material for: Modulation of transcription factor dynamics allows versatile information transmission
Source: Sci Rep. 2023 Feb 14;13:2652. doi: 10.1038/s41598-023-29539-3 (PMC9929046; doi:10.1038/s41598-023-29539-3)
Supplement: Supplementary file 1 — Supplementary Information. [file 41598_2023_29539_MOESM1_ESM.pdf]

Modulation of transcription factor dynamics allows versatile information transmission. Supplementary Note.

Alan Givré[1], Alejandro Colman-Lerner[2,3,*] and Silvina Ponce Dawson[1,*]
[1]*Departamento de Física, FCEN-UBA, and IFIBA, CONICET-UBA, (1428) Buenos Aires, Argentina*
[2]*Department of Physiology, Molecular and Cellular Biology, School of Exact and Natural Sciences, University of Buenos Aires, Buenos Aires, Argentina*
[3]*Institute of Physiology, Molecular Biology and Neurosciences, National Scientific and Technical Research Council (IFIBYNE-CONICET), Buenos Aires, Argentina*
[*] *Corresponding authors; emails: colman-lerner@fbmc.fcen.uba.ar, silvina@df.uba.ar*

In this Supplementary Note we show how the simple transcription model that is used in the paper can be derived from a more mechanistic description of the steps involved.

TRANSCRIPTION MODEL

All computations of the paper have been done using the model depicted in its Fig. 1. The core of the model corresponds to the very simple dynamical description of transcription introduced in [1]:

$$P_0 \underset{d_1}{\overset{k_1 \frac{[TF]^n}{[TF]^n+K_d^n}}{\rightleftharpoons}} P_1 \xrightarrow{k_2 \frac{[TF]^n}{[TF]^n+K_d^n}} \text{mRNA} \xrightarrow{d_2} \varnothing, \tag{1}$$

where $[TF]$ is the concentration of transcription factor in the nucleus; P_0 and P_1 are the probabilities, respectively, that the promoter is in the inactive or the active state; and mRNA is degraded at rate, d_2, and produced at the instantaneous rate $P_1(t)k_2[TF(t)]^n/(K_d^n + [TF(t)]^n)$. It is assumed that the protein produced up to a certain time, t, is proportional to the time integral of mRNA up to t. This model is presented in [1] as a simplified, analytically solvable version of the more detailed one:

$$P_0 \underset{\tilde{d}_1}{\overset{\tilde{k}_1[TF]}{\rightleftharpoons}} P_b \underset{\tilde{d}_2}{\overset{\frac{\tilde{k}_2[TF]^n}{[TF]^n+\tilde{K}_d^n}}{\rightleftharpoons}} P_1 \xrightarrow{\frac{\tilde{k}_3[TF]^n}{[TF]^n+\tilde{K}_d^n}} \text{mRNA} \xrightarrow{\tilde{d}_3} \varnothing, \tag{2}$$

also introduced in the same paper. Hansen and O'Shea present this more detailed model not as giving a mechanistic description of transcription but rather as the model, among the 12 they probed, that better reproduced their experimental observations. In particular, they argue that "having the transcription step be dependent" on $[TF](t)$, was "essential". This property, which also characterizes the simplified model (1), "means that transcription shuts off more or less immediately after" the TF "exits the nucleus", "that transcription requires" the TF "to be bound at the promoter". The authors also state that "an alternative way of accounting for this is to introduce another", transcriptionally active, promoter state that deactivates very quickly, but that they did not choose that option to minimize the number of free parameters of the model and avoid overfitting. We hereby provide a mechanistic description that justifies this dependence. We do it for the simplified model, but a similar approach can be followed with a more complicated kinetic scheme.

The first assumption that is apparent in the model (1) is that the binding/unbinding of the TF to the promoter occurs much faster than its activation/deactivation and much faster than the rate of transcription. Namely, TF molecules actually bind/unbind one by one, but if there is very strong cooperativity then there are mainly two "detectable" states in terms of binding: all the binding sites are either occupied or vacant:

$$P_u \underset{k_B}{\overset{k_F[TF]^n}{\rightleftarrows}} P_b, \tag{3}$$

where P_u and P_b represent the promoter with all its sites free or occupied, respectively. Now, the promoter (actually, the DNA molecule) can also be in different conformations. Here we consider that the promoter can be in two which we arbitrary call "active" and "inactive". There are two modeling options here: to assume that the transition to the active state only occurs when the TF is bound or that the promoter can exist in two possible conformations and that TF binding favors one of them. The latter is the approach introduced in the seminal paper by Monod, Wyman and Changeux (MWC) to describe allosterism in proteins [2]. MWC-like models have been used to describe transcription in eukaryotes, in particular, to account for nucleosome-mediated TF cooperativity [3] and to model the regulation of the *hunchback* gene by the TF, Bicoid, in *Drosophila melanogaster* embryos [4], among other examples. The rationale for using MWC models in transcriptional regulation stems from the fact that genomic DNA in eukaryotes, due to its interaction with nucleosomes and the different configurations that chromatin may adopt, can exist in a state that is inaccessible to TF molecules but that, once a TF binds by chance, a configuration is favored in which TF binding sites become readily available for other molecules [5]. Thus, within this framework we may think of two states ("tense" and "relaxed", in terms of the original MWC nomenclature or "active" and "inactive", as we have been using in this

note) between which the promoter can switch. We show in what follows how the simplified model (1) can be derived combining the MWC framework, the approximate binding scheme, (3) and parameter values such that the transition from the inactive to the active state occurs preferentially when TF molecules are bound to the promoter. This last condition is in accordance with recent results on transcription in living *Drosophila* embryos which show that DNA accessibility is not the result of thermal fluctuations but is catalyzed by the TFs [6].

Let us consider a promoter that can be in four possible states: inactive with all its n binding sites free, P_u; active with all its binding sites free, P_u^*; active with all its sites bound to TF molecules, P_b^*; inactive with all its sites bound to TF molecules, P_b. Thus, the kinetic scheme is:

$$\begin{array}{ccc} P_u^* & \underset{k_B^*}{\overset{k_F^*[TF]^n}{\rightleftharpoons}} & P_b^* \\ \beta_u \Big\uparrow\Big\downarrow \alpha_u & & \beta_b \Big\uparrow\Big\downarrow \alpha_b \\ P_u & \underset{k_B}{\overset{k_F[TF]^n}{\rightleftharpoons}} & P_b \end{array} \qquad (4)$$

This kinetic scheme must be combined with the equation describing mRNA production and degradation (which, as explained in the paper, we model stochastically in terms of the number of mRNA molecules, $X(t) \in \mathbb{N} \cup \{0\}$). Within the framework of this mechanistic model we consider that mRNA production proceeds with rate, $k_T P_b^*$, *i.e.*, only if the promoter is in state P_b^* ("active" and with TF bound). We can interpret this conditioning as a consequence of the interaction of DNA with nucleosomes or of chromatin configuration as described before. Under this assumption the equations ruling the change in X are:

$$X = N \xrightarrow{k_T P_b^*(t)} X = N + 1; \quad X = N \xrightarrow{d_2 X} X = N - 1, \qquad (5)$$

with $N = \{0, 1, 2 \cdots\}$. Even though the transitions between the various promoter states in (4) form a loop, we must consider that these transitions not only depend on the promoter interaction with TF molecules but also with other components (*e.g.*, nucleosomes). Thus, the detailed balance condition (*i.e.*, that the product of the rates around the loop in one direction should be equal to the product in the opposite direction) does not necessarily hold.

Let us now consider the kinetic scheme (4) and assume that TF binding/unbinding occurs on a faster timescale than promoter activation/deactivation and transcription. Using the notation, $P_b(t)$, $P_u(t)$, $P_b^*(t)$ and $P_u^*(t)$, to denote both the states and the probability that the promoter is in each of them at time t, the evolution equations on this fast scale are approximately given by:

$$\begin{aligned} \frac{dP_u}{dt} &\approx -k_F[TF]^n P_u + k_B P_b, \quad \frac{dP_b}{dt} \approx k_F[TF]^n P_u - k_B P_b, \\ \frac{dP_u^*}{dt} &\approx -k_F^*[TF]^n P_u^* + k_B^* P_b^*, \quad \frac{dP_b^*}{dt} \approx k_F^*[TF]^n P_u^* - k_B^* P_b^*, \end{aligned} \qquad (6)$$

so that

$$P_0 \equiv P_u + P_b \approx constant, \quad P_1 \equiv P_u^* + P_b^* \approx contant, \quad \text{on the fast timescale}, \qquad (7)$$

$P_0 + P_1 = 1$ and the various variables tend to the (quasi) equilibrium solution:

$$P_b(t) \approx \frac{[TF]^n}{K_{d,0}^n + [TF]^n} P_0(t), \quad P_u(t) = P_0(t) - P_b(t) \qquad (8)$$

$$P_b^*(t) \approx \frac{[TF]^n}{K_{d,1}^n + [TF]^n} P_1(t), \quad P_u^*(t) = P_1(t) - P_b^*(t), \qquad (9)$$

that actually varies on the slow timescale. In Eqs. (8)–(9), $K_{d,0} \equiv (k_B/k_F)^{1/n}$ and $K_{d,1} \equiv (k_B^*/k_F^*)^{1/n}$ are effective dissociation constants of the TF-promoter binding/unbinding reaction in the promoter inactive and active states, respectively. The "constants" in Eq. (7) evolve on the slow timescale, always keeping $P_0 + P_1 = 1$. When the evolution on the slow timescale is considered, Eqs. (8)–(9) hold instantaneously (all the variables adjust their values to a new, slowly changing, equilibrium, as expressed by the time dependence in these equations).

As already mentioned, we assume that mRNA production proceeds at a slower rate than binding/unbinding, so that it can be approximated by:

$$\text{rate of mRNA production} = k_T P_b^*(t) \approx k_T \frac{[TF]^n}{K_{d,1}^n + [TF]^n} P_1(t). \qquad (10)$$

We then need to determine how $P_1(t)$ varies on the "slow" timescale (with "slow" meaning "slower" than TF binding/unbinding).

According to scheme (4), the evolution of P_1 is determined by:

$$\frac{dP_1}{dt} = \frac{d}{dt}\left(P_u^* + P_b^*\right) = -\alpha_b P_b^* + \beta_b P_b - \alpha_u P_u^* + \beta_u P_u. \tag{11}$$

Inserting Eqs. (8)–(9) we obtain the evolution equation of P_1 on the slow timescale:

$$\frac{dP_1}{dt} = -\alpha_u P_1 - (\alpha_b - \alpha_u)\frac{[TF]^n}{K_{d,1}^n + [TF]^n}P_1 + \beta_b \frac{[TF]^n}{K_{d,0}^n + [TF]^n}P_0 + \beta_u \frac{K_{d,0}^n}{K_{d,0}^n + [TF]^n}P_0, \tag{12}$$

which can be solved given that $P_0 = 1 - P_1$. Eq. (12) describes the backward/forward transitions in the first step of the simplified model (1) provided that $|\alpha_b - \alpha_u| \ll \alpha_u$ and $\beta_u \ll \beta_b$. The latter inequality holds under the very reasonable assumption that the transition from the inactive to the active state of the promoter occurs with much higher probability per unit time if the promoter is bound to TF molecules than if it is not (*i.e.*, that the transition $P_b \to P_b^*$ occurs more often than $P_u \to P_u^*$). As already mentioned, this is in accordance with recent studies of transcription in living *Drosophila* embryos which concluded that DNA accessibility was catalyzed by the TFs [6]. The condition $|\alpha_b - \alpha_u| \ll \alpha_u$ holds if we assume that the relaxation from the active to the inactive state occurs approximately at the same rate regardless of whether TF molecules are bound to the promoter or not. Under these assumptions that could also be justified in the ability of the model (1) to reproduce the main experimental observations with a minimum number of parameters [1], Eq. (12) becomes:

$$\frac{dP_1}{dt} = -\alpha_u P_1 + \beta_b \frac{[TF]^n}{K_{d,0}^n + [TF]^n}P_0, \tag{13}$$

which corresponds to the backward/forward transitions in the first step of (1) if we identify $\alpha_u \approx \alpha_b = d_1$ and $\beta_b = k_1$. Eqs. (13) and (10) justify the "double dependence" of the transcription rate of the simple model (1) on $[TF]$ (through the transition from P_0 to P_1 and through the transcription rate, $k_T \frac{[TF]^n}{K_{d,1}^n + [TF]^n} P_1(t)$). The mechanistic description we are providing here shows that, for this double dependence to make sense, P_0 and P_1 must be considered as the probability that the promoter is inactive or active, respectively, including both situations with TF bound and unbound. The $[TF]$ dependence of the transition rate from P_0 to P_1 then arises because the elementary transitions of the mechanistic model (4) occur between substates of P_0 and P_1 whose probabilities can be approximated as $[TF]$-dependent fractions of P_0 and P_1. We may notice that both in (1) and in the approximate description of the mechanistic model, (4), given by Eqs. (10) and (13)), the cooperativity index, n, is the same for the transition probability from P_0 to P_1 and for the transcription rate. The dissociation constant, however, which is also the same in the model (1) is not necessarily equal according to the more mechanistic description. We can always argue that the best fit to the experiments of [1] is such that $K_{d,0} \approx K_{d,1} = K_d$. However, we can also reason that even if k_F and k_F^* and k_B and k_B^* might be different, it is not unlikely that the energy difference between the bound and the unbound states when the promoter is in its active or in its inactive conformation be similar. This implies that the dissociation constants which depend on this difference satisfy $K_{d,1} \approx K_{d,0} = K_d$. Under this last assumption the approximate description of the mechanistic model (4) given by Eqs. (10) and (13) is equivalent to that of the simple model (1).

[1] A. S. Hansen and E. K. O'Shea, Molecular Systems Biology **9**, 704 (2013), https://www.embopress.org/doi/pdf/10.1038/msb.2013.56, URL `https://www.embopress.org/doi/abs/10.1038/msb.2013.56`.

[2] J. Monod, J. Wyman, and J.-P. Changeux, Journal of Molecular Biology **12**, 88 (1965), ISSN 0022-2836, URL `https://www.sciencedirect.com/science/article/pii/S0022283665802856`.

[3] L. A. Mirny, Proceedings of the National Academy of Sciences **107**, 22534 (2010), https://www.pnas.org/doi/pdf/10.1073/pnas.0913805107, URL `https://www.pnas.org/doi/abs/10.1073/pnas.0913805107`.

[4] R. Phillips, J. Kondev, J. Theriot, and H. Garcia, *Physical Biology of the Cell, 2nd ed.* (Garland Science, Taylor & Francis Group, New York, 2012), ISBN 978-0815344506, URL `https://www.taylorfrancis.com/books/mono/10.1201/9781134111589/physical-biology-cell-rob-phillips-hernan-garcia-julie-theriot-jane-kondev`.

[5] S. Marzen, H. G. Garcia, and R. Phillips, Journal of Molecular Biology **425**, 1433 (2013), ISSN 0022-2836, allosteric Interactions and Biological Regulation (Part I), URL `https://www.sciencedirect.com/science/article/pii/S0022283613001617`.

[6] E. Eck, J. Liu, M. Kazemzadeh-Atoufi, S. Ghoreishi, S. A. Blythe, and H. G. Garcia, eLife **9**, e56429 (2020), ISSN 2050-084X, URL `https://doi.org/10.7554/eLife.56429`.
